# Effect of Low Concentrations of Lithium Chloride Additive on Cellulose-Rich Ultrafiltration Membrane Performance

**DOI:** 10.3390/membranes13020198

**Published:** 2023-02-05

**Authors:** Anastasiia Lopatina, Mohammadamin Esmaeili, Ikenna Anugwom, Mika Mänttäri, Mari Kallioinen-Mänttäri

**Affiliations:** Department of Separation Science, LUT School of Engineering Science, LUT University, P.O. Box 20, FI-53851 Lappeenranta, Finland

**Keywords:** lithium chloride, wood, membrane fabrication, ultrafiltration, 1-ethyl-3-methylimidazolium acetate, choline chloride, lactic acid

## Abstract

Various water treatment processes make extensive use of porous polymeric membranes. A key objective in membrane fabrication is to improve membrane selectivity without sacrificing other properties such as permeability. Herein, LiCl (0–2 wt.%) was utilised as a preforming agent in fabricating biomass-derived cellulosic membranes. The fabricated membranes were characterised by dope solution viscosity, surface and cross-sectional morphology, pure water flux, relative molecular mass cut-off (MWCO, 35 kDa), membrane chemistry, and hydrophilicity. The results demonstrated that at the optimum LiCl concentration (0.4 wt.%), there is an interplay of thermodynamic instability and kinetic effects during membrane formation, wherein the membrane morphology and hydrophilicity can be preferably altered and thus lead to the formation of the membrane with better rejection at no detriment to its permeability.

## 1. Introduction

Pressure-driven membrane filtration is universally utilised to treat wastewater in different fields. A wide selection of polymeric membranes allows precise tailoring of the treatment process, using particular advantages of a membrane’s characteristics to the process’s benefit [1,2,3]. Cellulose-based membranes are usually defined to be hydrophilic, biodegradable, and low-fouling, thus having an advantage over most petroleum-based polymeric membranes [4,5]. The use and fabrication of cellulose-based pressure-driven membranes represent the development of more environmentally intelligent technologies as it both meets the demand for renewable materials and provides reliable and highly efficient treatment of waste streams without secondary pollution [1,3,6]. The general challenge, however, in manufacturing cellulose membranes is finding the appropriate solvent system for cellulose dissolution and regeneration. Recently, the research has been focused on using ionic liquids (ILs), the class of solvents consisting of a mixture of solely ions with a melting point usually below 100 °C, and their different combinations with co-solvents [7,8,9,10]. The utilisation of ILs for cellulose and biomass dissolution and treatment has been consecutively studied over the last two decades [11,12,13,14]. Various ILs have been successfully utilised for biomass and cellulose dissolution, preparation of composite materials, and fabrication of cellulose-based membranes [4,15,16,17,18,19,20]. However, the performance of lab-made cellulose-based membranes is usually less effective compared to both lab-made petroleum-based polymeric membranes and commercial membranes [21]. Different strategies are utilised to improve the performance of lab-made cellulose-based membranes, including the variations in the used coagulation bath conditions, the choice of cellulose solvents, and the utilisation of additives [4,22,23].

Lithium chloride (LiCl) is a well-known pore-forming additive and has been abundantly tested for the production of polymeric membranes with higher permeance rates [22,24,25,26,27,28]. One of the reasons why lithium chloride is principally appealing as an inorganic salt additive for membrane casting solutions is that LiCl interacts strongly to form complexes with solvents frequently used for membrane preparation [27]. However, the reported concentrations of both the polymer and the additive were usually higher than 5 wt.%, thus raising concerns about the sustainability of LiCl utilisation, keeping in mind the scarcity of lithium resources [27,29,30].

LiCl has already contributed to the development of cellulose dissolution studies, being part of the LiCl/ dimethylacetamide (DMac) solvent system [31,32,33]. Some of the studies also report the positive effect of the addition of LiCl to ILs and dimethylsulfoxide (DMSO) on the dissolution of cellulose and biomass in general [14,34,35]. Though studies are reporting the effect of LiCl addition on the properties of cellulose acetate membranes, to the current knowledge, there is no study reporting the influence of LiCl over the performance of cellulose-based membranes, especially the ones that are made from partially purified biomass source [36].

The present study aims to investigate the effect of LiCl salt on the rheology of dope solutions containing 1-ethyl-3-methylimidazolium acetate ([Emim][OAc])–DMSO–biomass-derived cellulose and further its role on the morphology and performance of fabricated biomass-derived cellulosic membranes. The cellulose-rich membranes were prepared using the wet-phase inversion technique. The casting solution was prepared from birch biomass pretreated with deep eutectic solvent (DES) for partial delignification and consequently bleached for further purification of the cellulose fraction; the detailed procedure and its effect on the biomass composition can be found in the previous publication [37]. The treated biomass was dissolved in the [Emim][OAc]–DMSO–LiCl system. The LiCl concentration was kept below 2 wt.% to take advantage of LiCl being a suitable pore former and additive and study its effect on the membranes’ morphology and performance while bearing in mind the scarcity of LiCl.

## 2. Materials and Methods

### 2.1. Materials

Debarked birch chips (*Betula pendula*) with an average size of 5 × 1 × 0.1 cm were used as source material for all described operations. DES treatment was applied for partial delignification of the biomass. Utilised DES consisted of choline chloride (ChCl, CAS # 67-48-1, Merck KGaA, Darmstadt, Germany), acting as a hydrogen bond acceptor (HBA), and lactic acid (LAc, CAS # 79-33-4, Merck KGaA, Darmstadt, Germany), acting as hydrogen bond donor (HBD). Deionised (DI) water mixed with ethanol at a 1:9 ratio was used for washing residual DES from treated biomass. Consequent bleaching of biomass was completed using acetic acid (CAS # 64-19-7, Merck KGaA, Darmstadt, Germany) and sodium chlorite (CAS # 7758-19-2, Acros Organics, Geel, Belgium). Ionic liquid (95% 1-ethyl-3-methylimidazolium acetate; C_1_C_2_ImOAc, CAS # 143314-17-4, Iolitec Ionic Liquids Technologies GmbH, Heilbronn, Germany) mixed with dimethylsulphoxide (CAS # 67-68-5, Merck KGaA, Darmstadt, Germany) at a 2:8 mass ratio was used for the preparation of the casting solution.

Non-woven polyester was used as a support material for the membrane preparation and was taken from used reverse osmosis (RO) membranes and cleaned mechanically and chemically. CENTRA-R 60\120 system (Elga purification system, Veolia Water, Lane End, UK) was used to produce ultra-pure DI water (15 MΩ, 0.5–1 µS/cm), which was used for the washing process, preparation of water-based solutions, and as a non-solvent in the coagulation bath. Anhydrous LiCl (CAS # 7447-41-8, Merck KGaA, Darmstadt, Germany) was used as an additive. Polyethylene glycol (PEG, approx. M_w_ 35 000 g/mol, CAS # 25322-68-3, Merck KGaA, Darmstadt, Germany) was used to prepare a model solution for the membrane retention study.

### 2.2. Methods

#### 2.2.1. DES Treatment

For the partial delignification of the birch woodchips, ChCl and LAc were taken at a 1:9 mole ratio, respectively, and mixed at 100 °C until a clear homogeneous mixture formed. The DES treatment was performed at a 1:5 solid-to-liquid mass ratio for 18 h at constant heating at 105 °C. The treated pulp was filtered through a filter paper under vacuum and washed with an ethanol/water mixture at a 9:1 volume ratio. The DES-treated pulp was moved to an oven and dried at 50 °C for 24 h.

#### 2.2.2. Bleaching

The bleaching of dried pulp was performed with DI water, sodium chlorite and acetic acid taken at an 80 mL:1 g:0.5 mL ratio to each 2.5 g of biomass. Bleaching was performed at constant heating at 70 °C for 60 min with repeated stirring every 5–7 min to ensure even treatment of the pulp load. At the end of the reaction (when the pulp was almost white-coloured), the pulp was separated from the liquid and subsequently washed with water, ethanol, and acetone. Then the pulp was dried in the oven at 50 °C until the measured weight difference was less than 1%.

#### 2.2.3. Membrane Preparation

For the preparation of the casting solution, LiCl in various concentrations was dissolved in the mixture of [Emim][OAc] and DMSO. After the dissolution of the additive was completed, the DES-treated and bleached pulp was added to the solution in small portions up to 5 wt.-% concentration. The solution was continuously heated at 100 °C (Heidolph Instruments GmbH & CO., Schwabach, Germany) under a constant stirring rate of 200 rpm until the pulp dissolved.

Automatic Film Applicator L (BYK-Gardner, Geretsried, Germany) was used for membrane casting: a prepared solution was cooled down to room temperature, poured on a casting plate with attached polyester support, and spread on a flat surface by casting knife with 300 µm casting thickness and 50 mm/s speed. Immediately after casting, the casting plate was transferred into a water coagulation bath (0 °C), where it was kept for 24 h. After the coagulation bath, membranes were washed under the DI water current to guarantee the complete removal of the solvents from the membrane structure. Circular coupons of 0.0038 m^2^ area were cut for further use.

#### 2.2.4. Casting Solutions Viscosity Measurements

The viscosity of the casting solutions cooled down to room temperature was measured based on Stokes’ law using the falling sphere method performed in the vertical glass cylinder. The measurement of each solution was repeated five times, and the presented results are averaged values.

#### 2.2.5. Membrane Permeability and Retention Measurements

**Figure 1:**
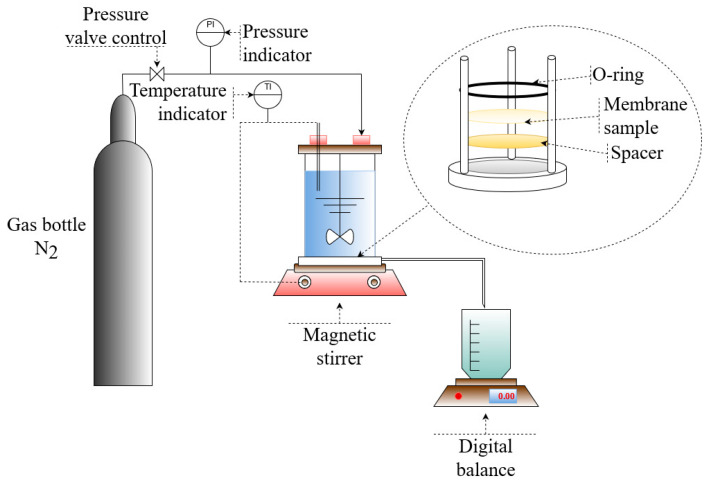
Amicon dead-end filtration system’s schematic configuration [38].

Amicon dead-end stirring cell equipment (Millipore, Temecula, CA, USA, Cat No.: XFUF07611; diameter of the stirring device: 60 mm) was used to measure the permeability and retention of the prepared membranes (see Figure 1). Prior to the filtration experiments, each membrane was compacted for 1 min at 1 bar, 2 min at 2 bars, 3 min at 3 bars, 4 min at 4 bars, and 20 min at 5 bars. The compaction process guaranteed the complete removal of the solvents used in the membrane manufacturing from the membrane pores. A permeate sample collected during the membrane compaction at 5 bars was analysed for total organic carbon (TOC) content to prove the absence of solvents’ remains.

For the determination of pure water permeability, membranes’ water flux was measured at 25 ± 0.5 °C at 1, 2, 3, and 4 bars of pressure, calculated using Equation (1), and plotted as a function of pressure:

(1) $$J=\frac{Q_{p}/100}{A\cdot \frac{t}{60}},$$

where J is the tested membrane’s flux (L/(m^2^·h)), *Q_P_* is the gravimetric flow of water permeating through the membrane (g/min), A is the area of the membrane sample (m^2^), and t is the time of collection of the permeate (min).

The retention of the produced membranes was studied with a model solution of PEG 35 kDa at a concentration of 300 ppm, which was filtered through the membrane samples at comparable flux values. All measurements were performed at 300 rpm stirring speed and 25 ± 0.5 °C. The feed, retentate, and permeate samples were collected and analysed for TOC content with a Shimadzu TOC analyser (TOC-L series, Japan). Equation (2) was used to calculate the retention values:

(2) $$R=\left(1-\frac{2\cdot C_{p}}{C_{f}+C_{r}}\right)\times 100,$$

where *C_p_*, *C_f_*, and *C_r_* are the TOC concentrations in the permeate, feed, and retentate (mg/L), respectively.

#### 2.2.6. SEM Analysis

The morphology of the prepared membranes was studied with the scanning electron microscope (Hitachi SU 3500, Tokyo, Japan) at an acceleration voltage of 1.5 kV in high vacuum conditions. For the analysis, the membrane samples were dried in Manual Freeze Dryer ALPHA 2-4 LDplus (Martin Christ GmbH, Osterode am Harz, Germany). The top surfaces of the membranes were analysed straight after freeze-drying. For the cross-sectional images, the narrow strips of membranes were cut and broken with two pairs of forceps under the liquid nitrogen to obtain a clean cut.

#### 2.2.7. Examination of the Chemical Structure of the Membranes

Frontier MIR/FIR Spectrometer (PerkinElmer Inc., Rodgau Germany) equipped with a diamond crystal was used to analyse the chemical structure of the membrane samples. The spectral range was 400–4000 cm^−1^, with a spectra resolution of 4 cm^−1^. Five points were measured from each membrane and averaged. All the spectra were processed for the graphical representation with ATR correction, baseline correction, and normalisation. The ratio of the non-normalised absorption bands A_1430_/A_899_ was used to calculate a lateral order index (LOI), as was proposed by Nelson and O’Connor [39,40].

#### 2.2.8. Contact Angle Measurements

The captive bubble method was applied for the evaluation of membrane hydrophilicity by a measure of static contact angle [41]. KSV CAM 101 equipment (KSV Instruments Ltd., Espoo, Finland) connected to a CCD camera (DMK 21F04, The Imaging Source Europe GmbH, Bremen, Germany) was used to measure contact angles. Each tested membrane was attached to a piece of glass with double-sided tape and submerged in DI water at room temperature. A U-shaped needle placed approximately 3–4 μL of air bubble volume on the membrane surface. Six points were measured from each membrane and averaged. The taken images were treated by curve fitting analysis with CAM 2008 software (Sydney Australia).

#### 2.2.9. Contact Angle Measurements

The water uptake capacity of the membrane samples was used to determine the membranes’ porosity. After being soaked in DI water for 24 h and carefully mopped with filter paper to remove the excess water, the wet membrane sample was weighed. Afterwards, the wet sample was dried in an oven at 60 °C for 24 h. The dry weight of the membrane sample was then measured until the sample weight became constant. The membrane porosity of the sample was subsequently calculated using Equation (3):

(3) $$\varepsilon =\frac{w_{w}-w_{d}}{\rho_{w}Al},$$

where ε is the membrane’s porosity, *w_w_* and *w_d_* are the weights of the wet and dry membrane samples (g), respectively, *ρ_w_* is the density of water at the temperature recorded during the measurement (23 °C) (0.997538 g/cm^3^), *A* is the area of the membrane samples (cm^2^), and *l* is the thickness of the membrane sample (cm), measured from the SEM images. The reported measurements are the averaged results from at least two membrane samples.

#### 2.2.10. Membrane Zeta Potential Measurement

The SurPASS electrokinetic Analyzer (Anton Paar GmbH, Graz, Austria) was used to measure the zeta potential of the membranes’ samples with an adjustable gap cell method and using 0.001 M KCl solution as a background electrolyte. Before the measurement, the membranes were kept in a fridge at approximately 4 °C for 24 h. The measurement started from pH 7.5, to which it was shifted by the addition of 0.1 M KOH solution and then automatically titrated to 2.7 using 0.05 M HCl solution as the analysis was carried on. The final value of the zeta potential was calculated automatically by SurPASS software (Anton Paar GmbH, Graz, Austria) based on the Helmholtz–Smoluchowski equation.

## 3. Results and Discussion

### 3.1. The Effect of LiCl Content on the Viscosity of Polymer Solution

**Figure 2:**
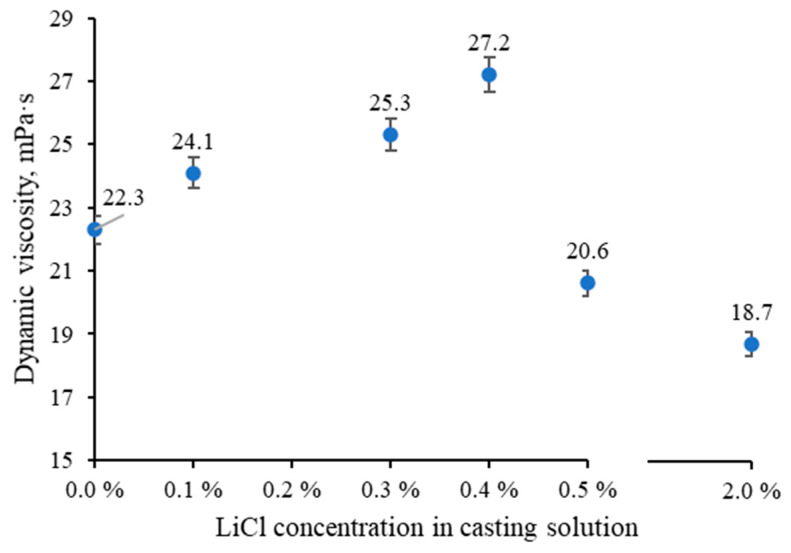
Dynamic viscosity values were measured with the falling sphere method at 23 °C.

Viscosity possesses a crucial role in controlling the formation kinetics of the membrane on both the skin layer and the sublayer level, which ultimately defines the performance of the fabricated membranes [42]. Figure 2 shows the effect of LiCl dosage on the viscosity of the casting solutions. Herein, the obtained viscosities align with the cellulose’s solution viscosities measured and reported in previous studies [43,44]. Although the role of LiCl in the changes happening to the casting solution viscosity has been highlighted in several studies, its effect on the viscosity of a complex interacting polymer solution mixture containing ionic liquid, DMSO, cellulose, and lignocellulose material has not been investigated to date [25,27,45,46]. As seen in Figure 2, the viscosity is sensitive to the amount of LiCl, and two distinct viscosity trends can be distinguished concerning the amount of added LiCl. A linear increase in viscosity with a successive increase in LiCl content up to 0.4 wt.% can be observed, after which it decreases by about 33% when the LiCl concentration reaches 2 wt.%.

The initial increase in viscosity can be attributed to the strong interactions between Li^+^ cation and electron donor groups within the solvent mixture [Emim][OAc]–DMSO, resulting in the reduced overall solvating power which in turn causes the formation of more prominent clusters of the polymer chains and thus increasing the viscosity [47,48,49,50]. It is worth mentioning that there is another possible interaction between the Li^+^ cation and hydroxyl groups in lignocellulose, which can aid swelling and dissolution of lignocellulose, especially hemicellulose and lignin when added to the ILs and DMSO solutions [14,34,35,51,52]. The decrease in dope solution viscosity at LiCl concentration above 0.4 wt.% could be related to the change of its interaction nature. It is known that alkali salts greatly influence intermolecular interactions when dissolved in DMSO solvent, destroying entanglement networks within molecular chains and, consequently, reducing viscosity [53].

### 3.2. The Effect of LiCl Content on the Morphology of the Membranes

#### 3.2.1. Physical Structure of the Membranes

**Figure 3:**
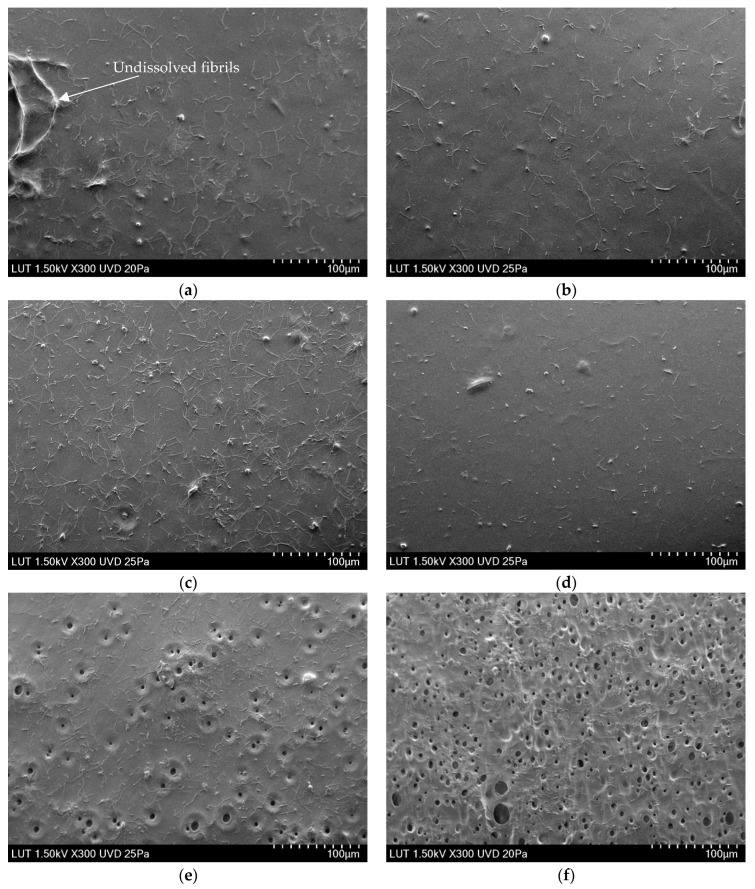
SEM images of membrane top surfaces taken via scanning electron microscope (Hitachi SU 3500, Japan) at an acceleration voltage of 1.5 kV in high vacuum conditions: (**a**) 0 wt.% LiCl, (**b**) 0.1 wt.% LiCl, (**c**) 0.3 wt.% LiCl, (**d**) 0.4 wt.% LiCl, (**e**) 0.5 wt.% LiCl, (**f**) 2 wt.% LiCl.

**Figure 4:**
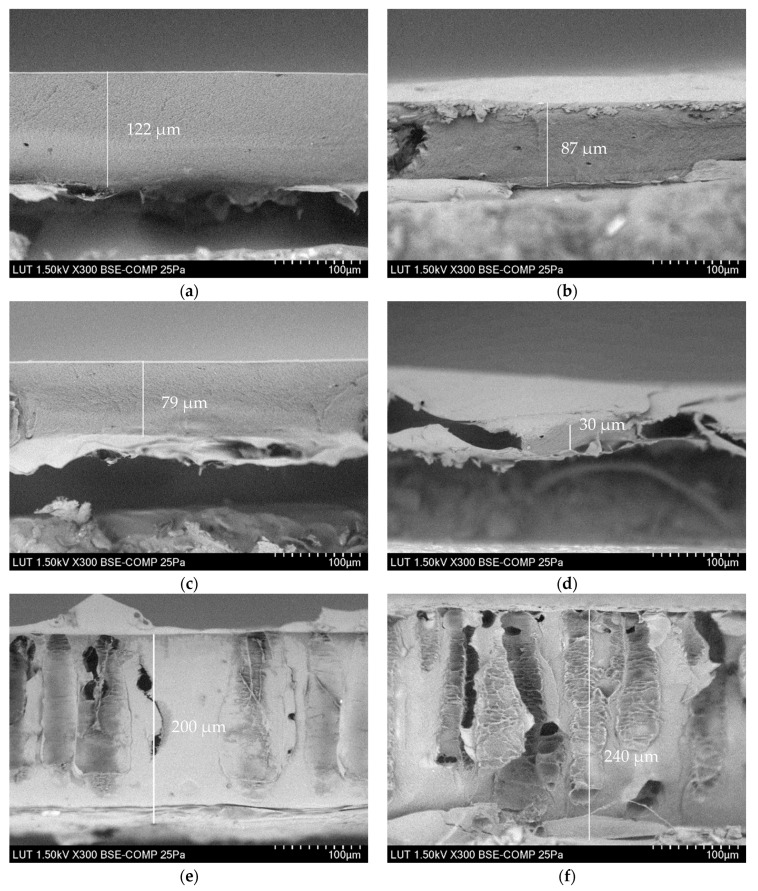
SEM images of membrane cross-sections taken via scanning electron microscope (Hitachi SU 3500, Japan) at an acceleration voltage of 1.5 kV in high vacuum conditions: (**a**) 0 wt.% LiCl, (**b**) 0.1 wt.% LiCl, (**c**) 0.3 wt.% LiCl, (**d**) 0.4 wt.% LiCl, (**e**) 0.5 wt.% LiCl, (**f**) 2 wt.% LiCl.

**Figure 5:**
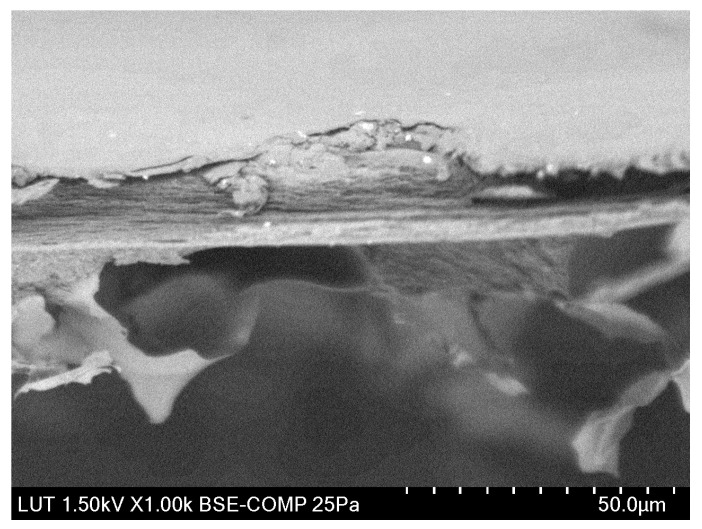
SEM image of 0.4 wt.% LiCl membrane’s cross-section taken via scanning electron microscope (Hitachi SU 3500, Japan) at an acceleration voltage of 1.5 kV in high vacuum conditions.

Figure 3, Figure 4 and Figure 5 show that the top layer morphologies and the cross-sections of membranes cast from LiCl-doped solutions are compared with the membrane cast from the unmodified solution. As can be seen (Figure 3), LiCl positively affects the dissolution process, resulting in a smaller number of visible fibrils and a smoother top layer. A positive effect of the presence of LiCl on the dissolution process in IL media has been reported for different kinds of polymers [14,34,51]. Up to 0.4% LiCl addition, membranes demonstrate smoothing of the top surface, which correlates well with the increase in the viscosity of the casting solutions and, thus, higher resistance of mass transfer during the phase inversion process and, eventually, delayed demixing and denser morphology (Figure 2). The 0.5 and 2 wt.% LiCl membranes show openings in the top layer, which agrees well with the lower viscosity of the respective casting solutions and the theory behind precipitation phenomena, where lower viscosity of the casting solution leads to quicker solvent-nonsolvent exchange process and thus more open membrane morphology [54].

#### 3.2.2. Thickness of the Membranes

In phase-inversion processes, the viscosity of the polymer solution can affect membrane thickness and interdiffusion of solvent and non-solvent [55,56]. According to general consensus, instantaneous demixing accelerates the precipitation of polymer chains, thereby increasing the membrane thickness, while delayed demixing delays precipitation completion [55]. As the viscosity of the casting solution increased continuously, from 0 wt.% to 0.4 wt.% LiCl concentration, the resulting membranes demonstrate similar sponge-like structures without macrovoids, which is a common feature of cellulose membranes precipitated from ILs’ solutions (Figure 4) [17]. It has also been reported that lithium-based additives positively affect the suppression of macrovoids formation [57]. As a result of the gradually increased casting solution’s viscosity and delayed demixing, the precipitation process finalised over a longer time, and thereby the thickness of the membrane decreased by ~75.4% (compared to the unmodified membrane) when the LiCl content reached 0.4 wt.%. At LiCl dosage above 0.4 wt.% in dope solution, the viscosity of polymer solution is drastically decreased (see Figure 2). At higher than 0.4 wt.% LiCl dosage, the thermodynamic miscibility of casting solution reduces and consequently promotes the kinetics of solvent outflux and non-solvent influx, which in turn increases the overall thickness of the membrane. As shown from the cross-section micrographs of modified membranes with 0.5 and 2% LiCl presented in Figure 3, the sponge structure gradually changes towards finger-like pores. This alteration can be attributed to the role of lower viscosity, reduction of thermodynamic stability of polymer solution and potential release of LiCl to the coagulation bath, which all lead to a faster exchange rate of solvent and non-solvent and formation of more open membranes [26,49,58].

The magnified cross-section of 0.4 wt.% LiCl membrane is depicted in Figure 5 to highlight its different morphology. It can be seen that only 0.4 wt.% LiCl membrane demonstrates a multi-layered structure, whereas the membranes with lower concentrations of LiCl show sponge-like morphologies and membranes with higher LiCl concentrations developed finger-like pores (see Figure 4). Although this kind of morphology alteration has been previously reported for more significant differences in salt concentrations, it might be concluded that in this particular complex system, even a more minor change in LiCl concentration possesses immense effect over the formed membranes’ morphology [59].

### 3.3. The Effect of LiCl Content on the Chemical Structure of the Membranes

**Figure 6:**
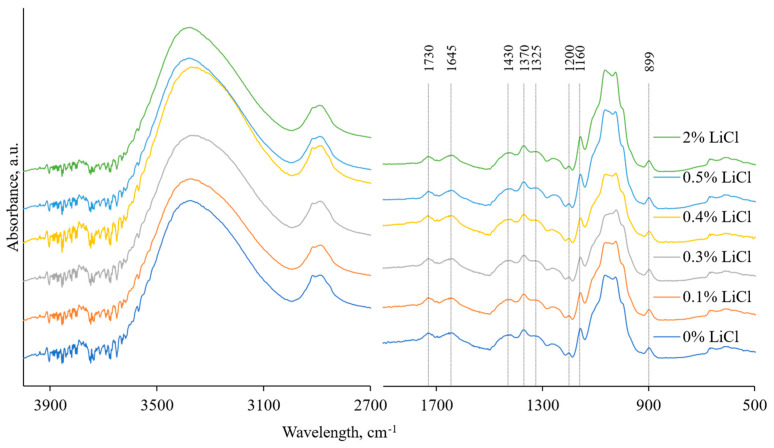
FTIR spectra of tested membranes were recorded using the Perkin Elmer Frontier spectrometer with a universal ATR module of diamond crystal at a resolution of 4 cm^−1^ in the absorbance mode.

FTIR spectra of unmodified membrane and modified with LiCl ones were recorded to evaluate the possible occurrence of chemical changes or interaction within the membrane structure (see Figure 6). Regardless of the added amount of LiCl, the spectra demonstrate the presence of the typical cellulose membrane’s peaks, the assignments of which can be found in the literature [37,60,61,62,63,64]. The peaks of practical interest are located at 1430 cm^−1^, representing in-plane symmetric bending characteristic of cellulose I_β_ crystal, and at 899 cm^−1^, characteristic of amorphous cellulose regions’ C-H deformation in β-glycosidic linkages. The absorbance values ratio at these peaks (A_1430_/A_899_) represents the amount of crystalline and amorphous cellulose in the membrane’s matrix or the lateral order index (LOI). The lower the value of LOI, the less ordered the cellulose structure and hence the lower the crystallinity [39,40,65]. The calculation shows a distinctive difference between LOI values of 0.1, 0.3, 0.5, and 2% LiCl membranes (all showing the LOI values within 1.21–1.23 interval) and 0 and 0.4% LiCl membranes, showing LOI of 1.33 and 1.28, respectively, indicating higher content of crystalline cellulose in those membranes. Although previously it has been stated that LiCl addition does not affect the crystallinity of the polymers [57], it might work differently for the cellulose regenerated from solutions with higher viscosity due to delayed demixing and improved orientation of the regenerated crystals and amorphous regions along the fibre axis [9].

### 3.4. The Effect of LiCl Content on Hydrophilicity and Zeta Potential of the Membranes

**Figure 7:**
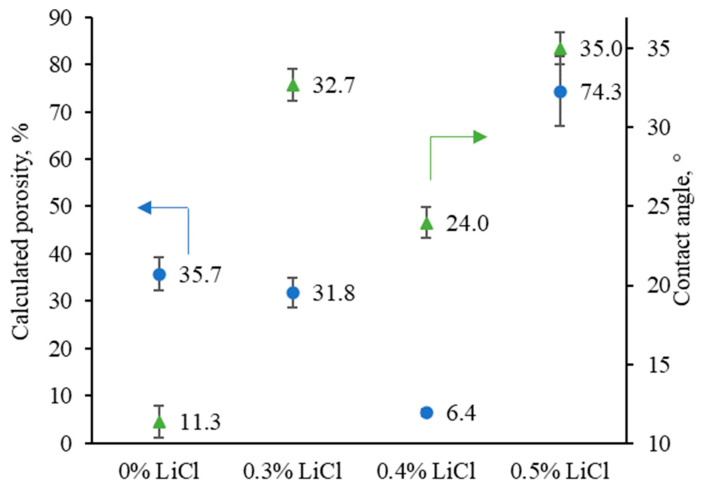
Variations in membranes’ porosity and contact angle as a function of LiCl additive concentration in the casting solution; the contact angle values of the prepared membranes were recorded using the captive bubble method with KSV CAM 101 equipment connected to a CCD camera.

Several factors influence the contact angle value, such as roughness, hydrophobicity or hydrophilicity, pore size and porosity, and distribution of pores. Figure 7 depicts changes in the membranes’ apparent contact angles and estimated porosities. All the tested membranes show the contact angle values typical for the regenerated cellulose membranes [5,52,66,67]. Generally, LiCl is reported to have a slight positive effect on the membranes’ surface hydrophilicity when coupled with petroleum-based polymeric membranes [47]. However, considering that the 0 wt.% LiCl membrane was very hydrophilic already before the LiCl addition (see. Figure 7), the addition of LiCl seems not to be having a similar effect on the hydrophilicity. It is worth stressing that the measured contact angle values are highly dependent on the surface’s morphology, which notably differs from one membrane sample to another and poses an effect over the measured values (see Figure 3).

**Figure 8:**
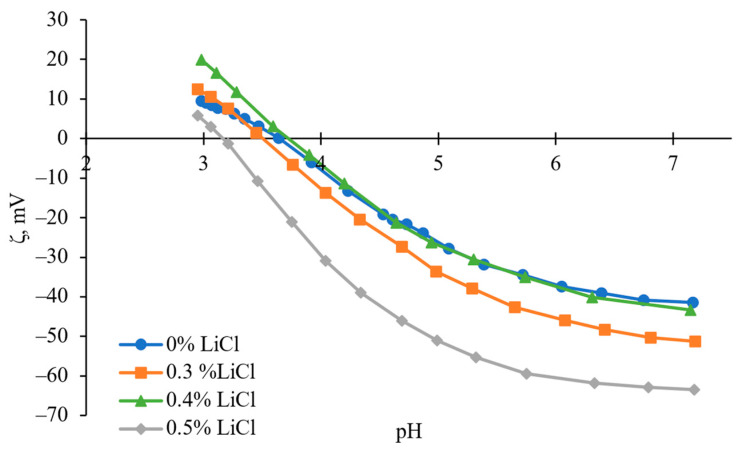
The zeta potential curves of prepared membranes were recorded using a SurPASS electrokinetic analyser with the adjustable gap cell method and using 0.001 M KCl solution as an electrolyte.

The zeta potential versus the pH of the reference and LiCl-modified membranes are presented in Figure 8. As can be seen, there is generally a negatively charged surface on the fabricated membranes over a wide pH range (4 to 7). Further confirmation of the speculation of changed interactions before and after 0.4 wt.% and induced solvent power by the addition of LiCl can also be noted in the isoelectric point shift of the modified membranes with 0.3 and 0.5 wt.% towards lower pH [68]. In general, with the increase in crystalline content in cellulose, the accessibility towards its charged group is more complicated compared to amorphous regions. As a result, it leads to cellulose with less negative surface charge [52]. Compared to the other membranes, 0 wt.% and 0.4wt.% LiCl ones showed a higher lateral order index, i.e., higher crystalline cellulose content, which can be considered a possible reason for their less negative surface charge. Another reason for increased zeta potential values can be found in the other membranes’ considerably higher overall porosity, including surface porosity. For example, the overall porosity of modified membranes with 0.3 and 0.5 wt.% increased by ~4.9 and ~11.6 times compared to 0.4 wt.% modified membrane. According to previously published reports, the increase in the accessibility of functional groups results in more negative net ζ-potential, which inevitably follows the formation of multiple openings when 0.5 wt.% LiCl is added (see Figure 3e) [68,69,70,71,72].

### 3.5. The Effect of LiCl Content on the Filtration Performance of the Membranes

**Figure 9:**
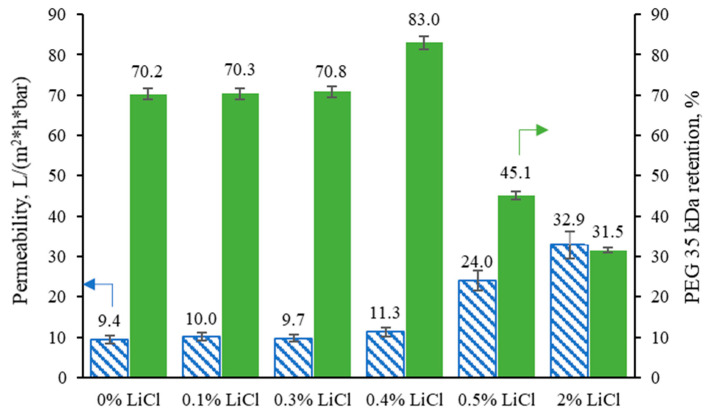
Pure water permeability values were measured at 1 bar, and PEG 35 kDa of the tested membranes was measured in an Amicon ultrafiltration cell at 25 °C and a mixing rate of approximately 300 rpm.

The performance of the prepared membranes as a function of the addition of LiCl is reported in Figure 9. The performance of fabricated membranes, i.e., flux and retention behaviours, are in accordance with the membranes’ morphology and the viscosity of the dope solutions. The pure water flux of the membrane was slightly increased with the increase in LiCl content up to 0.4 wt.%, which can be related to the reduction of membrane thickness (see Figure 4). Due to the dominant role of viscosity, particularly at the highest one (i.e., 0.4 % LiCl dosage, see Figure 2), the solvent/non-solvent mutual diffusion is hindered, and so-called delayed demixing takes place, resulting in the formation of denser structure (see Figure 7). Slow demixing results in nucleation after a specific time, which increases polymer concentration in the top layer. After that, nucleation occurs successively over a short period of time in the inferior layer. Hence, it can be said that slow demixing prevents the unrestrained growth of limited nuclei on the top layer, resulting in many small nuclei scattered throughout the film [73].

Consequently, the suppression of macrovoids and the formation of denser structure occurs, which can be considered a reason for the ~13% improvement of PEG 35 kDa retention for membrane fabricated at 0.4 wt.% LiCl content compared to the 0 wt.% membrane. At a concentration above 0.4 wt.%, the possible washing out of the salt from the membrane matrix and the viscosity reduction (see Figure 2) enhances the solvent/non-solvent exchange rate, i.e., instantaneous demixing. It thus leads to the formation of more open membranes (see Figure 3) with higher porosity (see Figure 7), which dominates a trade-off between higher permeability and worse selectivity.

## 4. Conclusions

In this work, cellulose-based ultrafiltration membranes were prepared from wood using a wet phase inversion casting method. Lithium chloride was chosen as a good pore-forming additive and added to the mixture of [Emim][OAc] and DMSO before adding DES-treated and bleached birch biomass. The concentration range for LiCl was kept low to study the effect of low concentrations and suggest the sustainable concentration of the additive. Based on the available literature, the expected outcome was an increase in membranes’ permeability. However, the results showed that in the mixture with wood-based biomass of complex composition and chosen solvents, the effect of LiCl over the membrane morphology and performance did not repeat the same trends as it did with other polymers. Even small changes in the amount of added LiCl were found to have an immense effect on the viscosity of casting solutions and the morphology of the formed membranes. The optimum concentration of the LiCl additive was found to be 0.4 wt.%, where an improvement of the separation efficiency by 13% is observed without the loss of permeability. In contrast, the further increase in LiCl dosage was impractical. The complexity of LiCl interactions with wood polymers chains, solvent, and co-solvent molecules might explain why LiCl shows different effects over the casting solution viscosity and consequently on the membrane’s morphology and performance depending on the concentration, where even the tiny alteration changes the entire outcome of LiCl presence.

## Data Availability

The data presented in this study are available upon request from the corresponding author.
